# Toxicity and outcomes after external beam irradiation for prostate cancer in patients with prior holmium laser enucleation of the prostate: Early experience

**DOI:** 10.1002/cnr2.1672

**Published:** 2022-07-05

**Authors:** Brady S. Laughlin, Gopi L. Narang, Scott M. Cheney, Mitchell R. Humphreys, Carlos E. Vargas, Sameer R. Keole, Jean‐Claude M. Rwigema, Steven E. Schild, William W. Wong

**Affiliations:** ^1^ Department of Radiation Oncology, Mayo Clinic Phoenix Arizona USA; ^2^ Department of Urology, Mayo Clinic Phoenix Arizona USA

**Keywords:** prostate cancer, urological oncology

## Abstract

**Purpose/Objectives:**

Holmium laser enucleation of the prostate (HoLEP) is commonly performed in patients with significant bladder outlet obstruction. However, there are few reports on the toxicity of external beam irradiation (RT) for prostate cancer in patients after prior HoLEP. In this study, we evaluate the side effects and treatment outcomes of RT after HoLEP.

**Materials/Methods:**

Eighteen patients who had HoLEP and subsequently received RT for prostate cancer were included. Data collected included patient and disease characteristics, urinary function, and radiation dose. Acute and late urinary (GU) and gastrointestinal (GI) side effects were evaluated. Disease control and survival rates were calculated using Kaplan–Meier method.

**Results:**

Median follow‐up was 18 months (range: 4–46 months). Median prostate volume was 107 ml before HoLEP and 24 ml after HoLEP. Median International Prostate Symptom Score (IPSS) was 17 (range: 5–32) before HoLEP. Median decline in IPSS score after HoLEP was 7 (range: −2–21). On uroflow study, peak flow rate, and post‐void residual were significantly improved after HoLEP. After radiation, peak flow rate and average flow rate showed a decline but remained significantly improved compared to pre‐HoLEP measurements. Maximum acute Common Terminology Criteria for Adverse Events (CTCAE) adverse events were 12 grade 1 and 3 grade 2 for GU, and 3 grade 1 for GI, respectively. Maximum late adverse events were 13 grade 1 and 2 grade 2 for GU, and all grade 0 for GI, respectively. At last follow‐up, there were 8 grade 1 and 1 grade 2 late GU, and 3 grade 1 late GI adverse events, respectively. There was no significant increase in urinary incontinence after RT compared to before RT. The 18‐month biochemical control, local control, distant control rates were 78%, 94%, and 80%, respectively.

**Conclusions:**

Patients who received RT as definitive treatment for prostate cancer after prior HoLEP had low risk of serious acute and late side effects. HoLEP can be safely performed and should be considered in patients with significant bladder outlet obstruction and large prostate volume before RT.

## 1 INTRODUCTION

Holmium laser enucleation of the prostate (HoLEP) is a surgical procedure which can alleviate urinary outlet obstruction and obstructive symptoms in the setting of benign prostatic hyperplasia or prostate cancer when medical therapy is unsuccessful.^1^ Other surgical options include transurethral resection of the prostate (TURP) and open prostatectomy. In comparison to open prostatectomy or TURP, HoLEP is associated with less morbidity.^2^, ^3^ The efficacy and safety of HoLEP has been demonstrated in multiple randomized trials evaluating HoLEP versus TURP or open prostatectomy.^4^, ^5^, ^6^, ^7^ HoLEP is unique in that it is a prostate size independent modality which is minimally invasive.^8^ It is especially suited for complex patients, specifically those on anticoagulation or with massive prostate glands.^9^, ^10^, ^11^ As studies continue to demonstrate superior functional outcomes of HoLEP compared to TURP, evidence supports its use as standard of care for bladder outlet obstruction secondary to BPH.^12^ There is limited data regarding the role of HoLEP in patients who have lower urinary tract symptoms (LUTS) secondary to prostate cancer.

While TURP has been associated with more severe genitourinary (GU) toxicity after external beam irradiation (RT) for prostate cancer, there are few studies exploring the overall impact of HoLEP on urinary function when RT is delivered after the procedure.^13^ We sought to understand how HoLEP impacts the toxicity profile in patients with prostate cancer receiving radiation. A retrospective review of patients with prior HoLEP who subsequently received RT for prostate cancer was performed to evaluate the toxicity and outcome of treatment. Here we report the early experience after a median follow‐up of 18 months.

## 2 METHODS

Between May 2008 and February 2020, we identified 18 patients who received RT for prostate cancer after HoLEP at our institution. After obtaining institutional review board approval, medical records of these patients were reviewed.

Patients received either intensity modulated radiotherapy (IMRT) or proton beam therapy. Prior to radiation, fiducial markers were placed in the prostate. Five patients also had spacer gel placement. This was followed by CT simulation and MRI for treatment planning. Radiation target volumes encompassed the prostate and seminal vesicles. IMRT was used in 7 and proton beam therapy was used in 11 patients. Several dose‐fractionation regimens were used, including conventional fractionation for 14 patients (75.6–82 Gy/1.8–2 Gy per fraction), moderate hypofractionation for 3 patients (60–70 Gy/2.5–3 Gy per fraction), and ultra‐hypofractionation for 1 patient (35Gy/7Gy per fraction). Sixteen patients received androgen deprivation therapy (ADT) with radiation treatment. The median duration of ADT was 18 months.

Patient and disease characteristics, and radiation treatment details were evaluated (Table 1). A baseline assessment of urinary symptoms was performed using IPSS score. Uroflow study was performed to evaluate bladder emptying prior to and after HoLEP as well as prior to and after radiation treatment. Acute and late gastrointestinal (GI) and GU toxicity was evaluated using the Common Terminology Criteria for Adverse Events (CTCAE). GU adverse events included frequency, urgency, obstruction, and dysuria. Grade 1 refers to mild symptoms that do not need any intervention. Grade 2 refers to moderate symptoms which require non‐invasive medical intervention, for example, use of alpha‐blockers such as tamsulosin, or NSAIDs such as ibuprofen. Patient follow‐up after treatment was every 3–6 months. Acute toxicity was defined as adverse events occurring within 3 months of completion of radiation treatment and late toxicity was defined as events occurring any time afterwards. Biochemical recurrence was defined as a rise in the prostate‐specific antigen (PSA) of 2 ng/ml above the nadir.^14^ Overall survival time was calculated from completion of radiation to last‐follow‐up.

**TABLE 1:** Patient and treatment characteristics of those treated with HoLEP prior to radiation therapy

Patient and treatment characteristics	
Age at diagnosis	75 (range 64–80)
Age at HoLEP	74 (range 64–79)
Clinical T‐stage	
1b	2 patients (11%)
1c	6 patients (33%)
2	3 patients (17%)
3a	6 patients (33%)
4	1 patient (6%)
Grade group	
2	1 patient (6%)
3	6 patients (33%)
4	4 patients (22%)
5	6 patients (33%)
NCCN risk group	
2	6 patients (33%)
3	12 patients (67%)
Diagnosis method	
Biopsy	14 patients (78%)
HoLEP	4 patients (22%)
PSA at cancer Dx (median)	8.1 ng/ml (range 4.2–91.2)
Prostate volume before Holep (median)	107 ml (range 29–168)
Prostate volume after Holep (median)	23 ml (range 14–74)
HoLEP planned prior to XRT	
Yes	11 patients (61%)
No	7 patients (39%)
Time from HoLEP to XRT (months) (median)	5.5 months (range 2.5–117)
Pre‐XRT PSA (median)	0.2 ng/ml (range 0.1–6)
Radiation technique	
Protons	11 (61%)
IMRT	7 (39%)

Abbreviations: HoLEP, holmium laser enucleation of the prostate; IMRT, intensity modulated radiotherapy; PSA, prostate‐specific antigen.

Statistical analysis was performed using JMP statistical program (JMP®, Version 14, SAS Institute Inc., Cary, NC, 1989–2019). *T* test was performed to compare the uroflow study measurements (voided volume, peak flow rate, average flow rate, post void residual volume) before and after HoLEP, as well as before HoLEP and after RT. Kaplan Meier method was used to calculate biochemical control rates.

## 3 RESULTS

### 3.1 Patient and disease characteristics

Median age of patients was 74 (range: 64–79) at the time of HoLEP. Prostate cancer diagnosis was made as an incidental finding at the time of HoLEP in four patients with symptoms of bladder outlet obstruction. Fourteen patients had prostate biopsies confirming presence of cancer prior to HoLEP. For 11 patients, HoLEP was performed prior to RT due to significant urinary obstruction. Median PSA at time of cancer diagnosis was 8.2 ng/ml (range: 4.2–91 ng/ml). Six patients had intermediate‐risk and 12 patients had high‐risk prostate cancer per NCCN guideline. Gleason grade groups were 2, 3, 4, 5 in 1 (6%), 6 (33%), 4 (22%), and 6 (33%) patients, respectively. One (6%) patient had prostatic ductal adenocarcinoma.

### 3.2 Prostate volume and urinary flow

Median prostate volume was 107 ml (range: 29–180 ml) prior to HoLEP. After HoLEP, median prostate size decreased to 24 ml (range: 14–74 ml). Median IPSS score was 17 (range: 5–32) before HoLEP. Uroflow parameters before and after HoLEP were listed in Table 2. There was improvement in urinary symptoms after HoLEP, with median decline in International Prostate Symptom Score (IPSS) of 7 (range:−2–21). Median voided urine volume after HoLEP increased to 164 ml from 94 ml (*p* = .30). Median peak urinary flow rate improved from 7 ml/s before to 16 ml/s after HoLEP (*p* = .014). Median average flow rate improved from 4 to 9 ml/s (*p* = .029). Median post void residual decreased from 96 ml before to 22 ml after HoLEP (*p* = .075).

**TABLE 2:** Urinary flow studies before and after HoLEP

	Before HoLEP (range)	After HoLEP (range)	Post‐RT^a^ (range)	*p* value before HoLEP versus after HoLEP	*p* value before HoLEP versus post‐RT
Voided volume (median)	94 ml (42–650)	164 ml (48–415)	99 ml (28–210)	0.30	0.49
Peak flow (median)	7 ml/s (2–15)	16 ml/s (2–35)	10.6 ml/s (4–20)	0.014	0.047
Average flow (median)	4 ml/s (2–15)	8 ml/s (2–20)	6.5 ml/s (2–10)	0.029	0.045
Post void residual (median)	96 ml (3–637)	22 ml (0–106)	31 ml (3–90)	0.075	0.080

Abbreviations: HoLEP, holmium laser enucleation of the prostate; RT, toxicity after external beam irradiation.

^a^ Eleven patients underwent uroflow testing after completion of RT.

### 3.3 Toxicity of radiation treatment

The median time between HoLEP and RT was 5.5 months (range: 2.3–117 months). Although HoLEP improved bladder emptying of patients, seven patients had mild incontinence after the procedure prior to RT initiation. Median IPSS score before RT initiation was 8 (range: 2–14). The median first IPSS score after radiation was 9 (range: 2–23) at a time of 4.9 months (range: 3–7.4 months) post treatment. The median IPSS score remained at 9 (range: 1–23) after a median follow‐up of 13.5 months. Eleven patients underwent uroflow study after completion of radiation therapy. The median voided volume, peak flow rate, average flow rate, and post void residual were 99 ml (range 28–210), 10.6 ml/s (range: 4–20), 6.5 ml/s (range: 2–10), and 31 ml (range: 3–90), respectively. The peak flow rate and average flow rate after RT were lower compared to post‐HoLEP measurements but remained significantly better than the pre‐HoLEP measurements (*p* = .047 and .045, respectively). Adverse events after radiation are tabulated in Table 3. The maximum acute CTCAE GU toxicity following RT was grade 1 for 12 (66%) patients, grade 2 for 3 (17%) patients. The maximum acute CTCAE GI toxicity following RT was grade 1 for 3 patients. With longer follow‐up beyond 3 months, the maximum late CTCAE GU toxicity was grade 1 for 13 (72%) patients and grade 2 for 2 (11%) patients. Maximum late CTCAE GI toxicity was grade 1 for 3 patients. At last follow‐up, 8 (44%) patients had grade 1, and 1 (6%) had grade 2 late GU side effects, while 3 had grade 1 late GI side effects, respectively. Bladder control in these patients did not worsen significantly after RT. At last follow‐up, eight patients (44%) had some urinary incontinence, as compared to seven patients before RT, but only culminated in four patients using pads and one using diaper. The median follow‐up in patients with urinary incontinence was 16 months (15–42 months).

**TABLE 3:** Acute and late toxicity following radiation in prostate cancer patients who had undergone HoLEP

IPSS	
IPSS prior to HoLEP	Median:17 (5–32)
IPSS after HoLEP, before radiation therapy	Median: 8 (2–14)
IPSS at 5 months after radiation	Median: 9 (2–23)
IPSS at 13.5 months after radiation	Median: 9 (1–23)

Adverse events after RT	Acute	Late	Last follow‐up
Max GU toxicity number of patients			
Grade 1	12 (67%)	13 (72%)	8 (44%)
Grade 2	3 (17%)	2 (11%)	1 (6%)
Max GI toxicity number of patients			
Grade 1	12 (67%)	3 (17%)	3 (17%)
Grade 2	3 (17%)	0	0

Abbreviations: GU, genitourinary; HoLEP, holmium laser enucleation of the prostate; IPSS, International Prostate Symptom Score; RT, toxicity after external beam irradiation.

### 3.4 Cancer treatment outcomes

**FIGURE 1:**
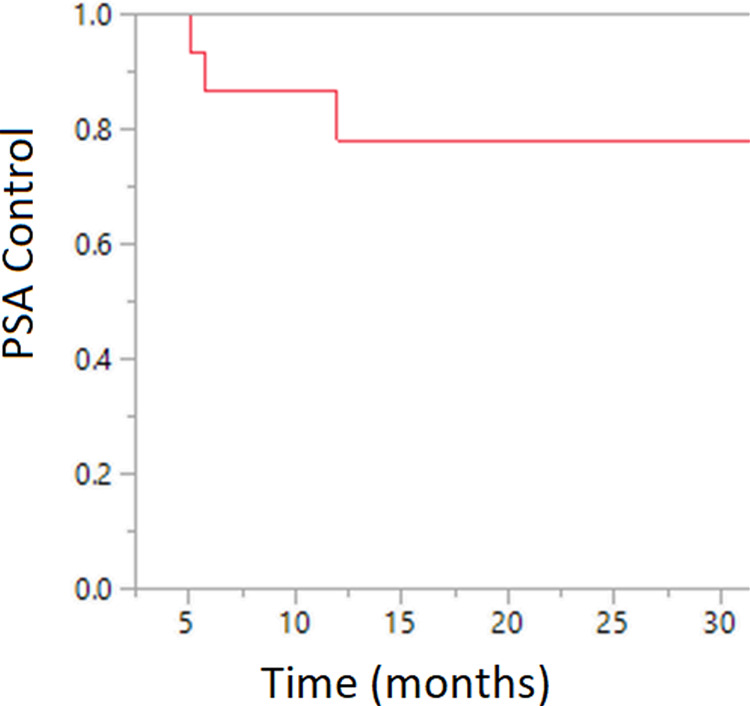
Kaplan Meier curve demonstrating biochemical control after radiation therapy

Median follow‐up was 18 months (range: 4–46 months). At 18 months, the biochemical control (Figure 1), local control, distant control rate was 78%, 94%, and 80%, respectively. At last follow‐up, one patient had developed local relapse and four patients had distant metastasis. The median time to the development of metastatic disease in these patients was 5.7 months. One patient died of disease at 2.4 years. Overall survival was 100% at 2 years and 75% at 3 years.

## 4 DISCUSSION

In men with significant benign prostatic hyperplasia and lower urinary tract symptoms, several surgical procedures are available to provide relief of bladder outlet obstruction, including TURP, open prostatectomy and HoLEP. HoLEP has established itself to be the treatment of choice for these patients because of its durability, low morbidity, and ability to treat prostates of all sizes. Studies have demonstrated the subjective and objective improvement in voiding parameters after HoLEP, and these advantages are even more amplified in large (>80 g) prostates.^2^, ^9^, ^15^ Compared to monopolar TURP, patients have shorter length of hospital stay, lower likelihood of readmission, and durable long‐term relief of urinary obstruction after HoLEP.^16^, ^17^

HoLEP has the potential benefit to alleviate significant LUTs. In a study of 552 patients with symptomatic BPH, Elzayat et al. reported significant improvement in voiding, with a 200% improvement in peak flow rate and I‐PSS at 1 year post‐operatively.^8^ Additionally, significant improvements have been reporting in voiding regardless of size of prostate.^8^, ^18^ Tamalunas et al. recently reported that men with prostates larger than 120 cc, LUTS significantly improved after HoLEP and there was no difference in postoperative outcomes compared to men with smaller prostates.^18^ Even in men over 80 years old, HoLEP can be performed and provide clinical benefits. In a study of 487 patients, the improvement in urinary function was seen in all age groups (<70 years, 70–79 years, and >80 years), with no difference in complication rates.^19^ While generally well tolerated, HoLEP has side effects. Becker and colleagues reported acute postoperative complications of clot retention in three patients (4.8%) and one patient required blood transfusion. Late complication included urethral stricture in one patient (1.6%) and bladder neck stenosis in two patients (3.2%), requiring urethrotomy and bladder neck resection.^20^ However, the study did not provide more details on the urinary side effects in patients who received radiation after HoLEP, except that one of two patients who developed bladder neck stenosis had received RT.^20^ There is also a risk of urinary incontinence after HoLEP. In our study, seven patients (39%) had some incontinence after HoLEP, before initiation of RT. After RT, eight patients (44%) reported some urinary incontinence, but only four patients reported using pads, and one using diaper. Becker et al also reported that patients who received RT after HoLEP had higher risk of incontinence.^20^ In patients who had HoLEP and managed by surveillance, 86% remained continent, while only 59% of those who received RT remained continent, with 23% requiring the use of 1 pad and 14% requiring 2 pads/day.^20^ Although the standard of care for intermediate‐risk and high‐risk prostate cancer includes radiation plus hormone therapy, HoLEP may serve a role in patients with low risk prostate cancer. Hagmann et al. reported the outcomes of 413 men diagnosed with prostate cancer by TURP and transrectal ultrasound (TRUS) guided biopsy.^21^ In comparison to men who underwent TURP, men diagnosed by TRUS guided biopsy had significantly higher reclassification, defined as pathological progression of tumor, (25% at 3.6 years vs. 25% at 11.2 years) while on active surveillance (*p* < .001).^21^

In patients who undergo a surgical procedure for BPH, incidental prostate cancer may be found in up to 12% of patients.^22^, ^23^ Many of these patients have low‐risk disease and can be managed with active surveillance or watchful waiting. However, some patients have higher risk prostate cancer and require definitive treatment. There are also patients who have significant pre‐existing BPH before the diagnosis of their prostate cancer. For these patients, a surgical procedure to relieve their bladder outlet obstruction prior to definitive radiation therapy is indicated to minimize the risk of significant radiation urinary side effects. TURP is often performed in this setting for smaller prostates (35–80 ml).^24^ Using a Surveillance Epidemiology and End Results registry data base, Krupski et al. reported that 9.3% of patients with prostate cancer underwent TURP.^25^ TURP performed before RT has been associated with increased urinary side effects.^25^ In a study by Devisetty et al., 71 patients with history of TURP received RT for prostate cancer. Grade 2 or higher acute GU toxicity occurred in 41% of patients, and the rate was higher in patients who underwent more than 1 TURP (73% vs. 31%).^13^ Late grade 3 or higher GU toxicity was more common in patients with TURP compared to those without TURP (16% vs. 4%, respectively). The risk ratio for a late grade 3 or higher GU toxicity after TURP was 2.87. In another study, late grade 2 or higher urinary incontinence rate after external beam irradiation was 2% for patients who had history of TURP, compared to 0.2% for those without history of TURP.^26^ There is a dearth of published data on the toxicity of radiation therapy in patients who have a history of HoLEP. In contrast to published data on TURP before radiation, our study demonstrated that the GU toxicity after RT in patients with history of HoLEP was low and acceptable, with no significant increase in risk of urinary incontinence.

In our study, patients had significant improvement in bladder emptying after HoLEP. Median IPSS was 17 (range: 5–32) before HoLEP, and the median decline in IPSS was 7 (range: −2–21) after the procedure. At 13.5 months after completion of radiation therapy, the median IPSS was 9, which remained better compared to prior to HoLEP. Uroflow study also demonstrated improvement in the urinary flow and decrease in postvoid residual. While the uroflow parameters after RT were not as good as after HoLEP, peak flow rate and average flow rate after RT remained significantly better than before HoLEP. Our findings were similar to those reported by Becker et al. In that study, 62 patients with diagnosis of prostate cancer underwent HoLEP, followed by RT in 22 patients (median dose of 72Gy), palliative treatment in 19, and surveillance in 21 patients.^20^ At a median follow‐up of 27 months, they reported improvement in urinary function, with median IPSS declining from 18.5 to 4.5, peak flow rate increasing from 9 to 18 ml/s, and post‐void residual decreasing from 100 to 0 ml. Thus, HoLEP can alleviate bladder outlet obstruction before definitive RT for prostate cancer and minimize the risk of significant urinary side effects after definitive radiation therapy. The median IPSS score after HoLEP, before radiation therapy, was 8. There was no significant deterioration of urinary symptoms, with a median IPSS score of 9 at 13.5 months after radiation.

In our study of prostate cancer patients who have undergone radiation following HoLEP, there was 1 local failure and 4 distant failures at the time of last follow‐up, with 18‐month biochemical control, local control and distant control rate of 78%, 94% and 80%, respectively. A preponderance of high‐risk prostate cancer (12/18 patients) in our patient population could have contributed to such result.

This study is the only report that provides a detailed description of the early results of toxicity and outcomes in patients who received definitive RT for prostate cancer after prior HoLEP. The data indicates that RT was safe and well tolerated, with only grade 1 and 2 acute and late urinary toxicity. The findings are reassuring that HoLEP can be safely performed before RT if indicated in patients with significant BPH and LUTs.

This study has several limitations. It is a retrospective study with a small number of patients and relatively short follow‐up. Additionally, our study is limited by lack of a control group. It does not provide a direct comparison between patients who received and did not receive HoLEP prior to radiation. The patient cohort was also dominated by those with high‐risk prostate cancer. Analysis with larger number of patients and longer follow‐up are needed to confirm the findings.

## 5 CONCLUSION

This study demonstrates that HoLEP can be safely performed in patients who have significant bladder outlet obstruction and LUTS before definitive RT for prostate cancer. The acute and late urinary toxicity after RT is mild and improves with time. There is no significant increased risk of incontinence after treatment.

## AUTHOR CONTRIBUTIONS

**Gopi Narang:** Validation (equal); writing – original draft (equal). **Scott Cheney:** Validation (equal); writing – original draft (equal). **Mitchell R. Humphreys:** Validation (equal); writing – original draft (equal). **Carlos Vargas:** Validation (equal); writing – review and editing (equal). **Sameer Keole:** Validation (equal); writing – original draft (equal). **Jean‐Claude Rwigema:** Validation (equal); writing – original draft (equal). **Steven Schild:** Validation (equal); writing – original draft (equal). All authors were involved with conception and design, writing, and final approval of this study.

## CONFLICTS OF INTEREST

The authors have stated explicitly that there are no conflicts of interest in connection with this article.

## ETHICS STATEMENT

The Mayo Clinic Institutional Review Board approved this study and was conducted in accordance with the Declaration of Helsinki of 1975 (as revised in 1985).

## CONSENT STATEMENT

Given the retrospective nature of this series, informed consent was waived.

## Data Availability

The data that support the findings of this study are available from the corresponding author upon reasonable request.

## References

[1] Moody JA , Lingeman JE . Holmium laser enucleation of the prostate with tissue morcellation: initial United States experience. J Endourol. 2000;14(2):219‐223. 10772518 10.1089/end.2000.14.219

[2] Ibrahim A , Alharbi M , Elhilali MM , Aubé M , Carrier S . 18 years of holmium laser enucleation of the prostate: a single center experience. J Urol. 2019;202(4):795‐800. 31009288 10.1097/JU.0000000000000280

[3] Gilling P . Holmium laser enucleation of the prostate (HoLEP). BJU Int. 2008;101(1):131‐142. 18086107 10.1111/j.1464-410X.2007.07341.x

[4] Gilling PJ , Kennett K , Das AK , Thompson D , Fraundorfer MR . Holmium laser enucleation of the prostate (HoLEP) combined with transurethral tissue morcellation: an update on the early clinical experience: an update on the early clinical experience. J Endourol. 1998;12(5):457‐459. 9847070 10.1089/end.1998.12.457

[5] Montorsi F , Naspro R , Salonia A , et al. Holmium laser enucleation versus transurethral resection of the prostate: results from a 2‐center, prospective, randomized trial in patients with obstructive benign prostatic hyperplasia. J Urol. 2004;172(5 Pt 1):1926‐1929. 15540757 10.1097/01.ju.0000140501.68841.a1

[6] Gupta N , Sivaramakrishna KR , Dogra PN , Seth A . Comparison of standard transurethral resection, transurethral vapour resection and holmium laser enucleation of the prostate for managing benign prostatic hyperplasia of >40 g. BJU Int. 2006;97(1):85‐89. 16336334 10.1111/j.1464-410X.2006.05862.x

[7] Kuntz RM , Lehrich K . Transurethral holmium laser enucleation versus transvesical open enucleation for prostate adenoma greater than 100 gm: a randomized prospective trial of 120 patients. J Urol. 2002;168(4 Pt 1):1465‐1469. 12352419 10.1016/S0022-5347(05)64475-8

[8] Elzayat EA , Habib EI , Elhilali MM . Holmium laser enucleation of the prostate: a size‐independent new “gold standard”. Urology. 2005;66(5, Supplement):108‐113. 10.1016/j.urology.2005.06.006 16194716

[9] Humphreys MR , Miller NL , Handa SE , Terry C , Munch LC , Lingeman JE . Holmium laser enucleation of the prostate—outcomes independent of prostate size? J Urol. 2008;180(6):2431‐2435. discussion 5. 18930490 10.1016/j.juro.2008.08.019

[10] Agarwal DK , Large T , Stoughton CL , et al. Real‐world experience of holmium laser enucleation of the prostate with patients on anticoagulation therapy. J Endourol. 2021;35:1036‐1041. 33280490 10.1089/end.2020.0886

[11] Zell MA , Abdul‐Muhsin H , Navaratnam A , et al. Holmium laser enucleation of the prostate for very large benign prostatic hyperplasia (≥ 200 cc). World J Urol. 2021;39(1):129‐134. 32206890 10.1007/s00345-020-03156-5

[12] Michalak J , Tzou D , Funk J . HoLEP: the gold standard for the surgical management of BPH in the 21(st) century. Am J Clin Exp Urol. 2015;3(1):36‐42. 26069886 PMC4446381

[13] Devisetty K , Zorn KC , Katz MH , Jani AB , Liauw SL . External beam radiation therapy after transurethral resection of the prostate: a report on acute and late genitourinary toxicity. Int J Radiat Oncol Biol Phys. 2010;77(4):1060‐1065. 20045267 10.1016/j.ijrobp.2009.06.078

[14] Roach M 3rd , Hanks G , Thames H Jr , et al. Defining biochemical failure following radiotherapy with or without hormonal therapy in men with clinically localized prostate cancer: recommendations of the RTOG‐ASTRO Phoenix Consensus Conference. Int J Radiat Oncol Biol Phys. 2006;65(4):965‐974. 16798415 10.1016/j.ijrobp.2006.04.029

[15] Cornu JN , Ahyai S , Bachmann A , et al. A systematic review and meta‐analysis of functional outcomes and complications following transurethral procedures for lower urinary tract symptoms resulting from benign prostatic obstruction: an update. Eur Urol. 2015;67(6):1066‐1096. 24972732 10.1016/j.eururo.2014.06.017

[16] Briganti A , Naspro R , Gallina A , et al. Impact on sexual function of holmium laser enucleation versus transurethral resection of the prostate: results of a prospective, 2‐center, randomized trial. J Urol. 2006;175(5):1817‐1821. 16600770 10.1016/S0022-5347(05)00983-3

[17] Elzayat EA , Elhilali MM . Holmium laser enucleation of the prostate (HoLEP): long‐term results, reoperation rate, and possible impact of the learning curve. Eur Urol. 2007;52(5):1465‐1472. 17498867 10.1016/j.eururo.2007.04.074

[18] Tamalunas A , Westhofen T , Schott M , et al. Holmium laser enucleation of the prostate: a truly size‐independent method? Low Urin Tract Symptoms. 2022;14(1):17‐26. 34323002 10.1111/luts.12404

[19] Tamalunas A , Westhofen T , Schott M , et al. The clinical value of holmium laser enucleation of the prostate in octogenarians. Low Urin Tract Symptoms. 2021;13(2):279‐285. 33260275 10.1111/luts.12366

[20] Becker A , Placke A , Kluth L , et al. Holmium laser enucleation of the prostate is safe in patients with prostate cancer and lower urinary tract symptoms—a retrospective feasibility study. J Endourol. 2013;28(3):335‐341. 24147796 10.1089/end.2013.0432

[21] Hagmann S , Ramakrishnan V , Tamalunas A , et al. Two decades of active surveillance for prostate cancer in a single‐center cohort: favorable outcomes after transurethral resection of the prostate. Cancers (Basel). 2022;14(2):368‐379. 10.3390/cancers14020368 PMC8773913 35053530

[22] Hutchison D , Peabody H , Kuperus JM , et al. Management of prostate cancer after holmium laser enucleation of the prostate. Urol Oncol. 2020;39:297.e1‐297.e8. 10.1016/j.urolonc.2020.11.003 33221258

[23] Rivera ME , Frank I , Viers BR , Rangel LJ , Krambeck AE . Holmium laser enucleation of the prostate and perioperative diagnosis of prostate cancer: an outcomes analysis. J Endourol. 2014;28(6):699‐703. 24484284 10.1089/end.2014.0009

[24] Persu C , Georgescu D , Arabagiu I , Cauni V , Moldoveanu C , Geavlete P . TURP for BPH. How large is too large? J Med Life. 2010;3(4):376‐380. 21254734 PMC3019067

[25] Krupski TL , Stukenborg GJ , Moon K , Theodorescu D . The relationship of palliative transurethral resection of the prostate with disease progression in patients with prostate cancer. BJU Int. 2010;106(10):1477‐1483. 20977594 10.1111/j.1464-410X.2010.09356.x PMC4507368

[26] Lee WR , Schultheiss TE , Hanlon AL , Hanks GE . Urinary incontinence following external‐beam radiotherapy for clinically localized prostate cancer. Urology. 1996;48(1):95‐99. 8693660 10.1016/s0090-4295(96)00085-4

